# IgG subclass-specific N-glycosylation differentiates HRCT subtypes in idiopathic inflammatory myopathies-associated ILD

**DOI:** 10.3389/fimmu.2025.1696126

**Published:** 2025-11-03

**Authors:** Tong Wu, Yanhong Li, Yingying Ling, Yubin Luo, Yinlan Wu, Jing Zhao, Lu Cheng, Chunyu Tan, Yong Zhang, Yi Liu

**Affiliations:** ^1^ Department of Rheumatology and Immunology, Laboratory of Rheumatology and Immunology, West China Hospital, Sichuan University, Chengdu, China; ^2^ Institute of Immunology and Inflammation, Frontiers Science Center for Disease-related Molecular Network, West China Hospital, Sichuan University, Chengdu, China; ^3^ Department of Pulmonary and Critical Care Medicine, State Key Laboratory of Respiratory Health and Multimorbidity, West China Hospital, Sichuan University, Chengdu, China; ^4^ West China Lecheng Hospital, Sichuan University, Boao, Hainan, China

**Keywords:** idiopathic inflammatory myopathies, interstitial lung disease, immunoglobulin G, N-glycosylation, glycoproteomics, nonspecific interstitial pneumonia, organizing pneumonia

## Abstract

**Introduction:**

Idiopathic inflammatory myopathies (IIMs) frequently involve interstitial lung disease (ILD), a major contributor to morbidity and mortality. However, the immunological heterogeneity across radiologic ILD subtypes remains poorly defined. This study aimed to explore whether subclass-specific IgG N-glycopeptides could distinguish high-resolution computed tomography (HRCT)-based ILD patterns in IIM patients.

**Methods:**

We analyzed plasma IgG subclass-specific N-glycopeptides in 145 IIM patients, including 98 with ILD (IIM-ILD), using intact glycopeptide mass spectrometry. Among IIM-ILD patients, 82 with available HRCT scans were classified into the three predominant subtypes: fibrotic nonspecific interstitial pneumonia (fNSIP, n=40), cellular NSIP (cNSIP, n=18), and organizing pneumonia (OP, n=24). A weighted multinomial logistic regression model was constructed using LASSO-selected glycopeptides and clinical variables.

**Results:**

Fifteen intact N-glycopeptides (IGPs) were quantified across all IIM patients. Compared to patients without ILD, IIM-ILD patients showed significant alterations in six IgG2-subclass IGPs. Crucially, distinct glycoform signatures were identified across the three HRCT subtypes: cNSIP was enriched in highly sialylated IgG2 forms; fNSIP showed elevated IgG1 and IgG3 glycoforms; and OP exhibited uniquely high IgG2-N5H4F1. These glycoforms correlated significantly with autoantibody profiles and clinical features. A multinomial logistic regression model integrating seven key IGPs and seven clinical variables achieved robust classification of the HRCT subtypes (macro-averaged AUC = 0.89).

**Conclusion:**

Subclass-specific IgG N-glycosylation profiles reflect the immunological heterogeneity underlying IIM-ILD. Integrating these glycan signatures with routine clinical data creates a strong model for distinguishing HRCT-defined endotypes, supporting their potential to improve disease classification and guide future mechanistic research in autoimmune-related ILD.

## 1 Introduction

Idiopathic inflammatory myopathies (IIMs) are a group of systemic autoimmune diseases characterized by heterogeneous clinical phenotypes and variable organ involvement (1). Interstitial lung disease (ILD) is among the most severe and life-threatening complications in IIMs, often determining long-term prognosis (2). High-resolution computed tomography (HRCT) enables the categorization of IIM-ILD into distinct patterns, including fibrotic nonspecific interstitial pneumonia (fNSIP), cellular NSIP (cNSIP), and organizing pneumonia (OP), which differ in clinical course and therapeutic response (3, 4). However, these imaging-based classifications offer limited insight into the underlying immunopathology, and considerable overlap in radiological features can make accurate subtype identification challenging. This limitation highlights the need for additional biomarkers that can capture immune-mediated diversity and improve HRCT-based classification.

Glycosylation of immunoglobulin G (IgG) plays a key role in regulating immune responses by modulating Fc receptor binding and complement activation (5, 6). Recent studies have suggested that altered IgG glycosylation patterns are related to various autoimmune diseases, including those complicated by ILD, such as anti-synthetase syndrome (ASS) (7–9). These studies suggest that glycosylation profiles can reflect disease-specific immune pathways. However, whether IgG glycosylation differs among HRCT-defined IIM-ILD subtypes remains unknown.

We hypothesized that subclass-specific IgG N-glycopeptides, when integrated with clinical features, could assist in distinguishing HRCT-based immunological endotypes in IIM-ILD patients. In this study, we profiled intact IgG N-glycopeptides across subtypes and built a glyco-clinical model to assess their added value beyond conventional serological and imaging markers, aiming to refine endotype-based classification and better capture disease heterogeneity.

## 2 Materials and methods

### 2.1 Patient subjects and sample collection

**Figure 1:**
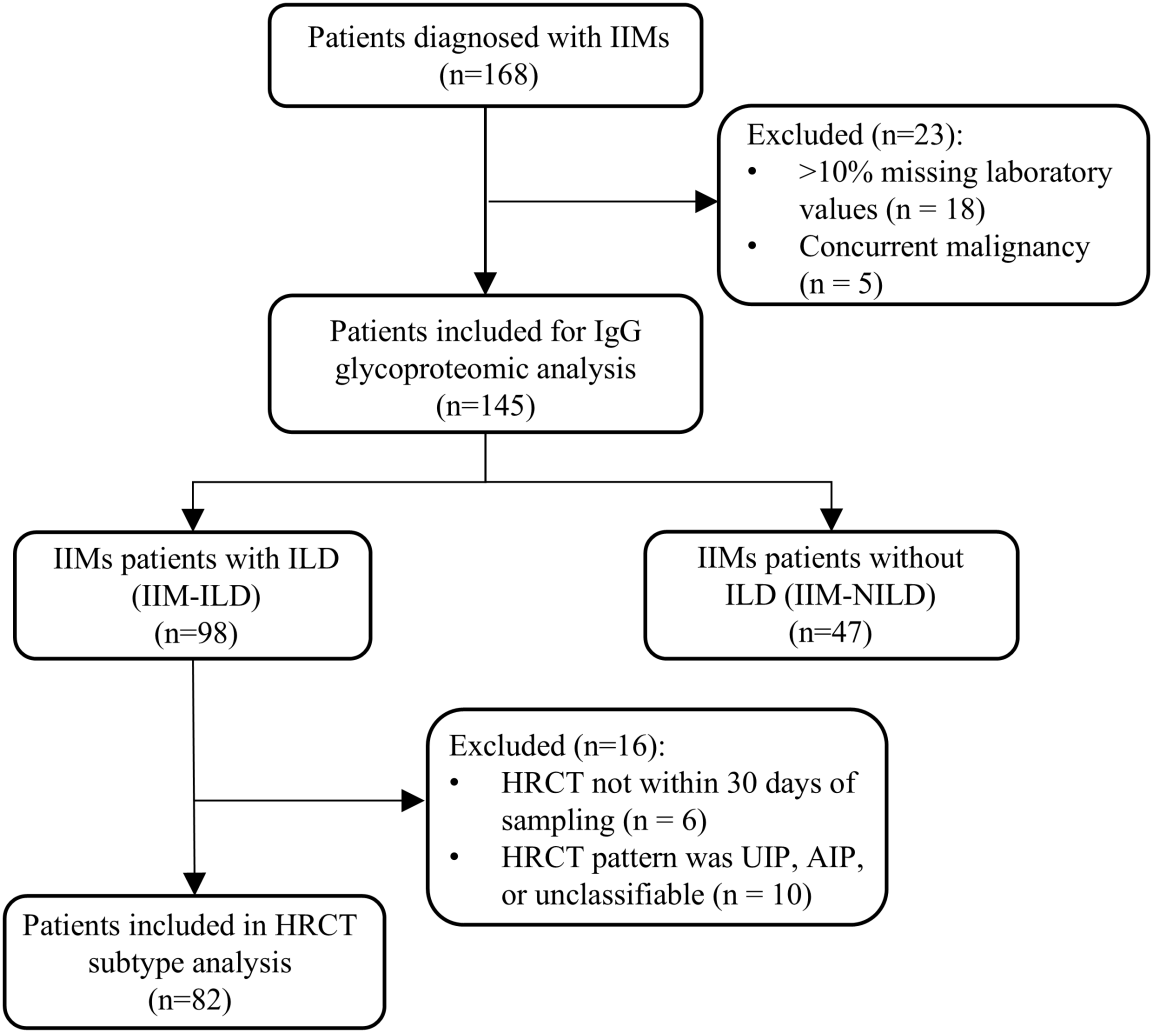
Flow diagram of patient enrollment. IIM, idiopathic inflammatory myopathies; ILD, interstitial lung disease; IIM-ILD, idiopathic inflammatory myopathies patients with interstitial lung disease; IIM-NILD, idiopathic inflammatory myopathies patients without interstitial lung disease; HRCT, high-resolution computed tomography; UIP, usual interstitial pneumonia; AIP, acute interstitial pneumonia.

A total of 168 patients diagnosed with IIMs were enrolled from the Department of Rheumatology, West China Hospital, Sichuan University, between January 2022 and December 2023. Diagnostic classification was standardized as follows: Amyopathic dermatomyositis (ADM) was diagnosed according to the criteria established by Euwer and Sontheimer (10); dermatomyositis (DM) and immune-mediated necrotizing myopathy (IMNM) were classified based on the 2017 EULAR/ACR guidelines (11); and anti-synthetase syndrome (ASS) was identified using the criteria proposed by Connors et al. (12). Two rheumatologists validated all diagnoses retrospectively. During data quality control, 18 patients with more than 10% missing laboratory values and five patients with concurrent malignancy were excluded. In total, the final cohort included 145 patients with IIMs. The detailed patient selection process is shown in **Figure 1**. The study protocol received approval from the Biomedical Research Ethics Committee, West China Hospital of Sichuan University (no. 341 in 2022), and all subjects provided written informed consent. Blood samples were collected the day after admission. Venous blood samples of 5 mL were collected in EDTA tubes, and then processed by centrifugation at 1,200 g for 10 minutes at 4 °C to separate the plasma. The plasma was stored at -80 °C until further analysis.

### 2.2 Clinical data collection and classification of ILD subtypes

Clinical data were systematically collected from electronic medical records upon initial hospitalization, including demographic characteristics, complications and comorbidities, clinical manifestations, laboratory findings, high-resolution computed tomography (HRCT) imaging features, and pulmonary function test (PFT) results. Muscle weakness was defined using the Medical Research Council muscle scale (score<5/5) (13). Autoantibody profiling included myositis-specific autoantibodies (MSAs: anti-ARS, anti-MDA5, anti-TIF1-γ, anti-Mi-2, anti-SAE-1, anti-NXP-2, anti-HMGCR, and anti-SRP) and myositis-associated autoantibodies (MAAs: anti-PM/Scl, anti-U1RNP, anti-Ro52, anti-SSA, anti-SSB, and anti-Ku). Anti-aminoacyl-tRNA synthetase antibodies (ARS) were specified as anti-Jo1, anti-EJ, anti-PL7, anti-PL12, and anti-OJ (1, 14, 15). All clinical parameters corresponded temporally with blood sampling timelines.

IIM patients with ILD (IIM-ILD) were diagnosed according to the international classification of connective tissue disease-associated interstitial lung disease (CTD-ILD) (16, 17). To align glycoproteomic sampling with imaging, only ILD patients with HRCT images taken within one month of blood sampling were included in the subtype classification. This resulted in 92 patients eligible for HRCT-based subtype analysis. Two experienced pulmonologists reviewed the HRCT images, and inter-observer agreement was measured using Cohen’s kappa (κ) statistic, resulting in a value of 0.856, indicating almost perfect agreement. Any differences in the initial classifications were addressed through discussion with a third senior pulmonologist to achieve a final consensus. The HRCT patterns were classified into cellular NSIP (cNSIP), fibrotic NSIP (fNSIP), organizing pneumonia (OP), usual interstitial pneumonia (UIP), and acute interstitial pneumonia (AIP) based on ATS/ERS guidelines for idiopathic interstitial pneumonia patterns (3).

### 2.3 Isolation, purification, and digestion of IgGs

All samples were randomly renumbered and processed in a blinded manner. The workflow for IgG isolation, purification, and enzymatic digestion followed established protocols (18). In brief, plasma samples were incubated with protein A/G affinity in binding buffer (20 mM Tris, 150 mM NaCl, pH 7.2) for 2 hours at 4 °C. After thorough washing, bound IgGs were eluted with 0.1 M formic acid, neutralized to physiological pH, and quantified via a bicinchoninic acid (BCA) protein assay (Thermo Fisher Scientific, USA). The purified IgGs were denatured at 95 °C for 10 minutes, reduced with 20 mM DTT at 56 °C for 30 minutes, and alkylated with 50 mM IAA at 25 °C for 30 minutes in the dark. After buffer exchange with 50 mM ammonium bicarbonate (pH 8.5) using 30 kDa filters, trypsin (0.5 μg) was added, and the mixture was digested at 37 °C for 2 hours. Peptides were quantified via a colorimetric peptide assay (Thermo Fisher Scientific, USA) at 480 nm.

### 2.4 LC-MS/MS analysis

For glycoproteomic analysis, digested peptides were analyzed via an Orbitrap Fusion Lumos mass spectrometer (Thermo Fisher Scientific, USA) equipped with a nanoelectrospray ionization source and coupled to an EASY-nLC 1200 HPLC system, as previously described (19). Peptides were dissolved in 0.1% formic acid (buffer A) and separated on a 20 cm ReproSil-Pur C18-AQ column (1.9 μm, 75 μm ID; Dr. Maisch) using a 30-minute gradient at a flow rate of 350 nL/min (5–12% B from 0–2 min; 12–22% B from 2–7 min; 22–32% B from 7–21 min; 32–90% B from 21–22 min; held at 90% B from 22–30 min), where buffer B was 0.1% formic acid in 80% acetonitrile. Fragmentation was performed using a hybrid EThcD and stepped-collision energy HCD (sceHCD) strategy, which provided higher spectral quality and greater identified depth. For quality control of mass spectrometry performance, an IgG mixture lysate prepared from pooled plasma of 30 IIMs patients was used as a quality control (QC standard. It was analyzed at the start of the run and then every 20 samples. This QC procedure enabled monitoring of instrument performance and potential inter-batch variation. The coefficient of variation (CV) of glycopeptide abundances across QC runs was assessed, and glycans with CV <15% were regarded as analytically stable and retained for further analysis.

### 2.5 Data processing and bioinformatic analysis

Intact N-glycopeptide (IGP) identification was conducted with glycopeptide identification via Byonic v3.10, using a custom human IgG database (UniProtKB entries IGHG1–4). Search parameters included a precursor ion tolerance of ±6 ppm and a fragment ion tolerance of ±20 ppm, allowing up to two missed tryptic cleavages. Fixed modifications included carbamidomethylation (C); variable modifications included methionine oxidation and N-terminal acetylation. A total of 182 annotated human N-glycans were specified as potential glycan modifications. To ensure confidence and reproducibility, glycopeptide identifications were filtered at a 1% false discovery rate (FDR), with only those achieving a Byonic score ≥200 and a minimum peptide length of six amino acids retained. All glycopeptide-spectrum matches (GPSMs) were manually inspected. Quantification was performed using PANDA (v1.2.5) in label-free mode by importing both raw MS files and identification results. These structures were labeled based on their composition: hexose (H), N-acetylhexosamine (N), fucose (F), and N-acetylneuraminic acid (A). A total of 15 IGPs were quantified: IgG1-N4H3F1, IgG1-N4H4F1, IgG2-N3H3F1, IgG2-N3H4F1, IgG2-N4H3F1, IgG2-N4H4, IgG2-N4H4F1, IgG2-N4H4F1A1, IgG2-N4H5F1, IgG2-N4H5F1A1, IgG2-N5H3F1, IgG2-N5H4F1, IgG2-N5H5F1, IgG3-N4H3F1, IgG3-N4H4F1. Relative abundances were normalized to the total intensity of all detected IgG Fc glycopeptides within each sample to enable cross-sample comparison. These normalized values were used for downstream analyses.

Continuous variables were summarized as mean ± standard deviation (SD) or median with interquartile range (IQR), depending on the data distribution. Categorical variables were reported as counts (%). For group comparisons, independent-samples t-tests, one-way ANOVA, or Kruskal–Wallis tests were applied based on the results of normality and homogeneity tests. *Post hoc* pairwise comparisons following the Kruskal–Wallis test were adjusted using Bonferroni correction, whereas Dunnett’s test was employed for multiple comparisons against a single reference group in ANOVA. Categorical variables were analyzed using the χ² test or Fisher’s exact test, as appropriate. All group comparisons were performed using GraphPad Prism 10, and two-sided p-values < 0.05 were considered statistically significant.

Before model construction, all continuous predictors were standardized using a z-score transformation to ensure comparability of effect sizes. To address class imbalance in the three HRCT subtypes (cNSIP, fNSIP, and OP), inverse class frequency weights were applied during model training. Subtype-specific glycopeptide features were selected using one-vs-rest LASSO logistic regression with 10-fold cross-validation to determine the optimal regularization parameter (lambda.min). Predictors with non-zero coefficients were retained for modeling. A multinomial logistic regression model was constructed to classify HRCT subtypes (cNSIP, fNSIP, and OP) using features selected from LASSO analysis. To evaluate multicollinearity among the predictors in the final multinomial model, we conducted a collinearity diagnostic by calculating the Variance Inflation Factor (VIF) for each variable. Model performance was evaluated using stratified 10-fold cross-validation, with metrics including accuracy, macro F1 score, class-specific F1 scores, and one-vs-rest area under the ROC curve (AUC). The macro-AUC was used to assess overall multi-class discrimination. Correlation analyses between glycoforms and clinical variables were performed using Pearson or Spearman’s rank correlation, as appropriate. All statistical analyses were conducted in R (version 4.5.0).

**Figure 2:**
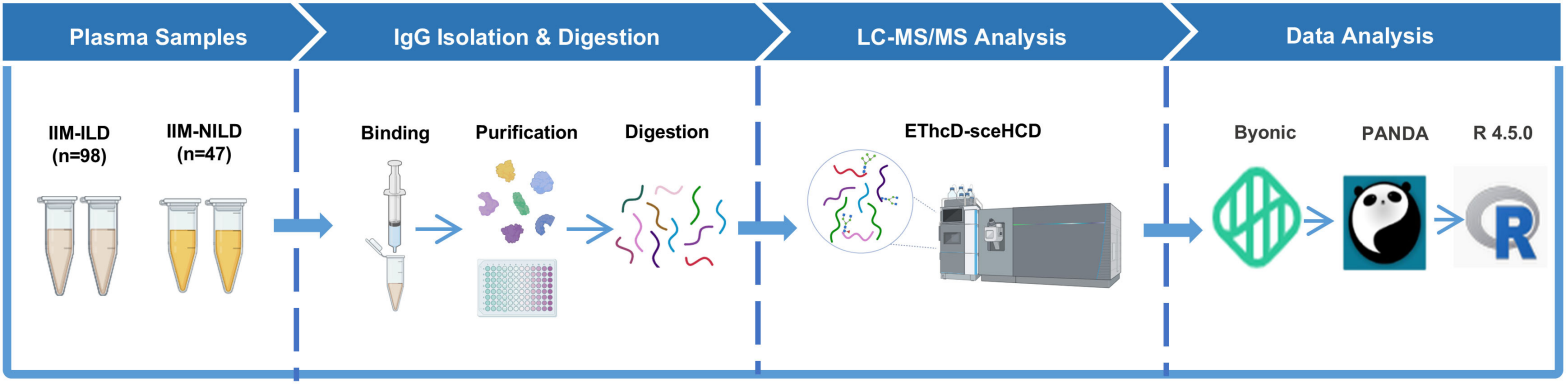
Schematic representation of the workflow for IgG intact N-glycopeptide analysis IIM-ILD, idiopathic inflammatory myopathies patients with interstitial lung disease; IIM-NILD, idiopathic inflammatory myopathies patients without interstitial lung disease.

The complete method flow was illustrated in **Figure 2**. All graphical visualizations, including boxplots, heatmaps, LASSO coefficient plots, confusion matrices, and ROC curves, were generated using GraphPad Prism 10 and R (version 4.5.0). The schematic representation of the workflow for IgG intact N-glycopeptide analysis was created with BioRender.com.

## 3 Results

### 3.1 Baseline characteristics of the IIM cohorts

A total of 145 patients diagnosed with IIMs were included in this study, comprising 98 patients with interstitial lung disease (IIM-ILD) and 47 without (IIM-NILD). Compared with IIM-NILD patients, patients with ILD presented significantly distinct clinical and immunological features (**Supplementary Table S1**). IIM-ILD patients were more frequently classified as having ASS or IMNM, whereas ADM was more common in the IIM-NILD group (p< 0.001). Muscle weakness was significantly less frequent in the IIM-ILD group compared to the IIM-NILD group (31.6% vs. 68.1%, p < 0.001). In contrast, autoantibodies associated with lung involvement were more common in the ILD group, including anti-MDA5 (53.1% vs. 25.5%, p = 0.002), anti-ARS antibodies (33.7% vs. 6.4%, p < 0.001), and anti-Ro52 (63.3% vs. 27.7%, p < 0.001). Laboratory findings revealed lower creatine kinase (CK) and lactate dehydrogenase (LDH) but higher peripheral neutrophil counts in IIM-ILD, indicating a distinct immunoinflammatory phenotype.

Among the 98 patients with ILD, 92 had available HRCT scans within 30 days of sampling. These patients were further classified into five HRCT subtypes based on ATS/ERS criteria: fNSIP (n = 40), cNSIP (n = 18), OP (n = 24), UIP (n = 2), and AIP (n = 4), with four unclassifiable. To ensure statistical power and focus on clinically representative patterns, subsequent analyses were restricted to the three predominant subtypes (fNSIP, cNSIP, and OP; n = 82). Clinical and serological characteristics differed significantly across these subgroups (**Table 1**). The cNSIP group was entirely female (100%) with the highest frequency of muscle weakness (61.1%, p = 0.008). The OP group had the shortest disease duration (median: 3.25 months, p = 0.005) and the highest frequency of pulmonary infection (62.5%, p = 0.043), alongside lower lymphocyte counts (p = 0.059). Distinct patterns were also observed in autoantibody profiles. Anti-MDA5 antibodies were enriched in cNSIP (66.7%) and OP (66.7%) versus fNSIP (35.0%, p = 0.018), whereas anti-ARS antibodies were most frequent in fNSIP (47.5%, p = 0.021). While most laboratory and pulmonary function indices showed no statistically significant differences, serum cTn levels were lowest in the OP group (p = 0.037). Medication patterns were broadly comparable among HRCT-defined subtypes (**Supplementary Table S2**). Anti-pulmonary fibrosis agents were prescribed in 22.50% of fNSIP and 12.50% of OP patients, but not in cNSIP. These findings highlight distinct immuno-clinical endotypes across HRCT patterns, supporting further subtype-specific glycosylation analyses.

**Table 1:** Clinical characteristics of IIM-ILD patients across HRCT-defined subtypes.

Variables	cNSIP (n=18)	fNSIP (n=40)	OP (n=24)	P value
Demographic data				
Female, n (%)	18 (100)	26 (65.00)	18 (25.00)	0.008
Age, years (mean, SD)	46.22 (14.46)	52.00 (9.35)	48.88 (11.16)	0.174
BMI, kg/m^2^ (mean, SD)	21.97 (3.00)	22.37 (3.13)	22.18 (3.41)	0.909
Disease duration, months (median, IQR)	7.00 (2.50, 21.00)	11.00 (4.50, 34.50)	3.25 (1.50, 9.50)	0.005
Smoking history, n (%)	-	6 (15.00)	3 (12.5)	0.273
Comorbidity, n (%)				
Hypertension	-	4 (10.00)	4 (16.67)	0.202
Diabetes	2 (11.11)	7 (17.50)	1 (4.17)	0.308
Cardiovascular disease	-	1 (2.50)	1 (4.17)	>0.99
Diagnosis, n (%)				
ADM	1 (5.56)	1 (2.50)	2 (8.33)	0.549
ASS	4 (22.22)	20 (50.00)	7 (29.17)	0.086
DM	7 (38.89)	13 (32.50)	9 (37.50)	0.868
IMNM	6 (33.33)	6 (15.00)	6 (25.00)	0.267
Clinical manifestations, n (%)				
Erythematous rashes	12 (66.67)	14 (35.00)	8 (33.33)	0.064
Heliotrope rash	5 (27.78)	2 (5.00)	-	0.008
Gottron’s sign	9 (50.00)	7 (17.50)	4 (16.67)	0.024
Mechanic’s hands	1 (5.56)	8 (20.00)	3 (12.50)	0.327
Muscle weakness	11 (61.11)	11 (27.50)	4 (16.67)	0.008
Arthritis/arthralgia	7 (38.89)	11 (27.50)	8 (33.33)	0.676
Pericardial effusion	4 (22.22)	7 (17.50)	3 (12.50)	0.639
Arrhythmia	2 (11.11)	3 (7.50)	4 (16.67)	0.476
Pulmonary infection	8 (44.44)	12 (30.00)	15 (62.50)	0.043
Laboratory findings				
ANA (+), n (%)	6 (33.33)	25 (62.50)	13 (54.17)	0.114
MAAs (+), n (%)	11 (61.11)	30 (75.00)	19 (79.17)	0.443
RO52 (+), n (%)	10 (55.56)	29 (72.50)	18 (75.00)	0.348
MSAs (+), n (%)	17 (94.44)	35 (87.50)	24 (100)	0.175
ARS (+), n (%)	2 (11.11)	19 (47.50)	9 (37.50)	0.021
MDA5 (+), n (%)	12 (66.67)	14 (35.00)	16 (66.67)	0.018
IMNM-associated MSAs (+), n (%)	2 (11.11)	1 (2.50)	1 (4.17)	0.333
CRP, ng/ml (median, IQR)	4.04 (1.76, 6.01)	4.31 (1.92, 9.69)	6.79 (2.96, 11.19)	0.186
CK, IU/L (median, IQR)	61.00 (36.75, 292.8)	64.00 (29.25, 479.5)	43.00 (24.50, 209.8)	0.462
LDH, IU/L (median, IQR)	294.5 (224.5, 482.8)	278.0 (236.8, 346.5)	264.5 (238.0, 355.8)	0.775
ALT, U/L (median, IQR)	52.00 (16.75, 89.50)	27.00 (18.50, 53.75)	43.00 (20.75, 81.5)	0.280
AST, U/L (median, IQR)	41.50 (22.25, 82.00)	23.50 (18.25, 43.50)	45.50 (21.50, 54.0)	0.074
MB, ng/ml (median, IQR)^†^	51.37 (27.62, 218.5)	41.74 (21.00, 214.0)	40.10 (22.62, 150.8)	0.881
CK-MB, ng/ml (median, IQR)^†^	50.80 (25.72, 157.3)	41.74 (21.00, 124.0)	35.70 (22.08, 105.1)	0.875
cTn, ng/L (median, IQR)^†^	28.60 (9.60, 231.5)	30.20 (14.45, 94.90)	11.95 (8.08, 27.13)	0.037
FER, (median, IQR)^††^	189.0 (72.90, 781.0)	614.0 (191.0, 884.0)	562.0 (352.5, 1080)	0.155
WBC, ×10^9^/L (mean, SD)	7.26 (3.91)	8.31 (3.72)	8.71 (4.52)	0.500
PMN, ×10^9^/L (mean, SD)	5.26 (3.16)	6.03 (3.23)	6.97 (4.17)	0.290
LYM, ×10^9^/L (mean, SD)	1.40 (0.87)	1.56 (0.80)	1.09 (0.55)	0.060
Pulmonary function^†††^				
FVC%, (mean, SD)	82.09 (22.32)	75.40 (15.83)	71.50 (19.84)	0.343
FEV1%, (mean, SD)	81.23 (25.55)	76.59 (16.03)	71.93 (21.02)	0.525
FEV1/FVC, (mean, SD)	82.82 (5.71)	85.13 (6.46)	85.25 (4.92)	0.443
DLCO%, (mean, SD)	68.81 (20.51)	61.84 (13.81)	63.66 (16.71)	0.393

†: MB, CK-MB, and cTn data are available for cNSIP (n=14), fNSIP (n=34), and OP (n=22); †† FER data are available for cNSIP (n=12), fNSIP (n=23), and OP (n=18); ††† pulmonary function data are available for cNSIP (n=15), fNSIP (n=33), and OP (n=9). IIM, idiopathic inflammatory myopathy; HRCT, high-resolution computed tomography; BMI, body mass index; cNSIP, cellular nonspecific interstitial pneumonia; fNSIP, fibrotic nonspecific interstitial pneumonia; OP, organizing pneumonia; ADM, amyopathic dermatomyositis; ASS, anti-synthetase syndrome; DM, dermatomyositis; IMNM, immune-mediated necrotizing myopathy; ANA, anti-nuclear antibody; MAAs, myositis-associated antibodies; MSAs, myositis-specific antibodies; ARS, anti-aminoacyl tRNA synthetase; MDA5, melanoma differentiation-associated gene 5; CRP, C-reactive protein; CK, creatine kinase; LDH, lactate dehydrogenase; ALT, alanine aminotransferase; AST, aspartate aminotransferase; MB, myoglobin; CK-MB, creatine kinase-MB isoenzyme; cTn, cardiac troponin; FER, ferritin; WBC, white blood cell count; PMN, neutrophil count; LYM, lymphocytes; FVC%, forced vital capacity; FEV1%, forced expiratory volume in 1 second; DLCO%, diffusing capacity of the lungs for carbon monoxide.

### 3.2 Differences in IgG N-glycopeptide profiles across HRCT-defined IIM-ILD subgroups

**Figure 3:**
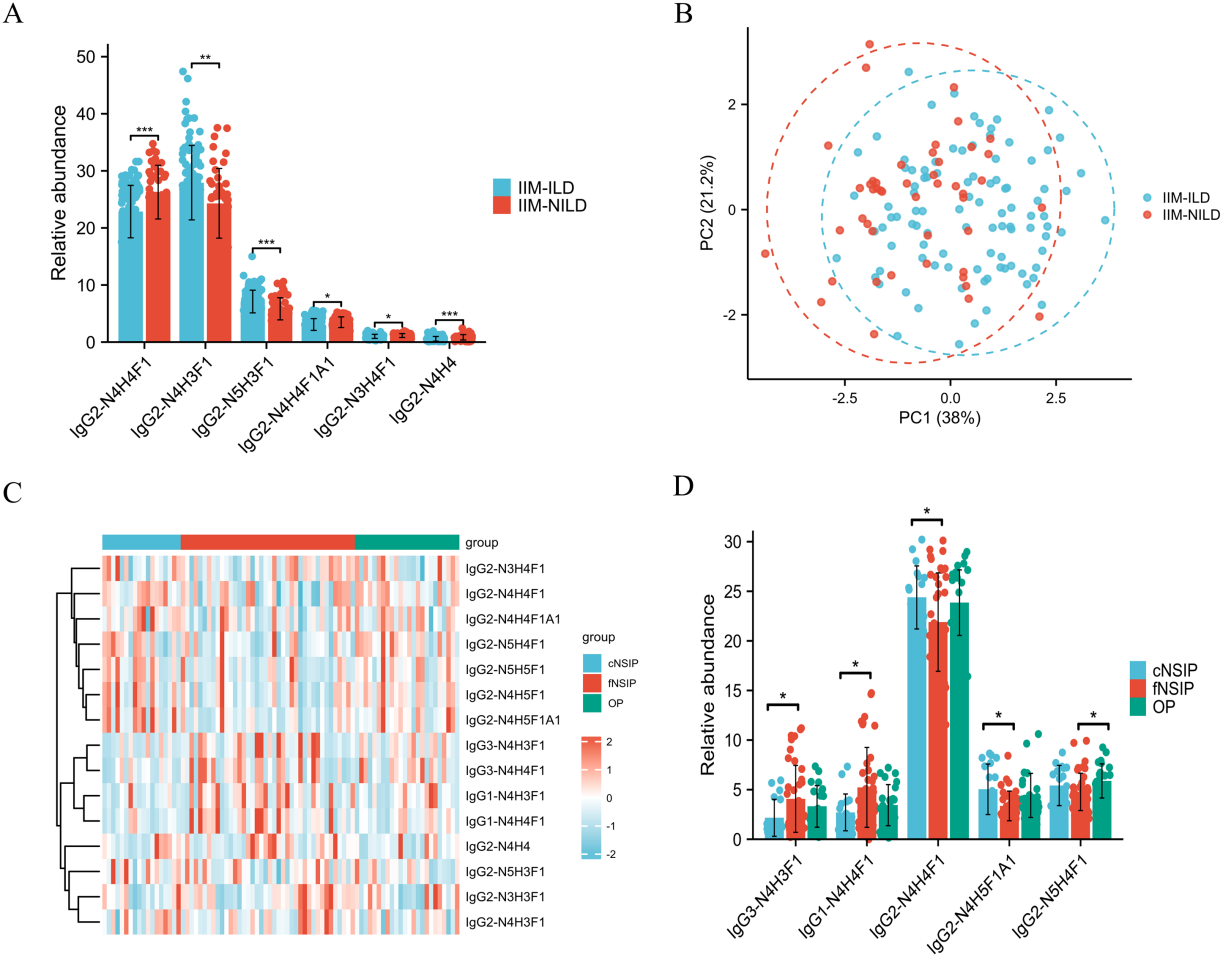
Distinct IgG N-glycopeptide profiles in IIM-ILD patients and across HRCT subtypes **(A)** Group comparison of six significantly different subclass-specific intact IgG N-glycopeptides (IGPs) between the IIM-ILD group and the IIM-NILD group. The independent-samples t-test or Mann-Whitney U test was used based on the results of normality and homogeneity tests. **(B)** The principal component analysis (PCA) plot demonstrates that six differential IGPs effectively differentiate between IIM-ILD and IIM-NILD patients. **(C)** Heatmap illustrating the distribution patterns of all 15 IGPs across three HRCT-defined subtypes of IIM-ILD. **(D)** Group comparison of five representative IGPs that showed significant differences among HRCT subtypes. One-way ANOVA, or Kruskal–Wallis tests were applied based on the results of normality and homogeneity tests, followed by *post hoc* pairwise comparisons with Bonferroni correction for multiple testing. cNSIP, cellular nonspecific interstitial pneumonia; fNSIP, fibrotic nonspecific interstitial pneumonia; OP, organizing pneumonia; N, N-acetylhexosamine; H, hexose; F, fucose; A, N-acetylneuraminic acid. Asterisk represents the value of p-value as follows: *p < 0.05, **p < 0.01, ***p < 0.001.

To explore glycosylation-related patterns in interstitial lung involvement, we analyzed the relative abundances of 15 subclass-specific IGPs and found significant differences between the IIM-ILD and IIM-NILD groups (**Supplementary Table S3**). At the global level, IIM-ILD patients exhibited a modest but statistically significant reduction in total sialylation (7.23% vs. 8.19%, p = 0.046). Among subclass-specific glycopeptides, six IGPs from the IgG2 showed significant differences (p < 0.05) (**Figure 3A**). IgG2-N4H3F1 (27.93% vs. 24.31%, p = 0.002), IgG2-N5H3F1 (7.11% vs. 5.83%, p < 0.001), and IgG2-N3H4F1 (1.00% vs. 1.15%, p = 0.023) were elevated in the IIM-ILD group, while IgG2-N4H4 (0.57% vs. 0.84%, p < 0.001), IgG2-N4H4F1 (22.86% vs. 26.27%, p < 0.001), and IgG2-N4H4F1A1 (3.08% vs. 3.48%, p = 0.025) were decreased. These IGPs effectively distinguished between IIM-ILD and IIM-NILD patients as shown in principal component analysis (PCA) plot (**Figure 3B**).

Building upon the observed glycosylation alterations, we further characterized glycoform patterns across HRCT-defined IIM-ILD subtypes and revealed five subclass-specific IGPs exhibiting significant subtype-specific differences (**Figures 3C, D**). In the cNSIP group, patients presented a relatively increased abundance of highly sialylated and galactosylated glycoforms. The levels of IgG2-N4H5F1A1 (5.04% ± 2.54%) and IgG2-N4H4F1 (24.38% ± 3.18%) were significantly higher compared to the fNSIP and OP group (p = 0.041 and p = 0.033, respectively). In contrast, the fNSIP group was characterized by elevated glycoforms primarily within the IgG1 and IgG3 subclasses. Specifically, the levels of IgG1-N4H4F1 (5.23% ± 4.03%) and IgG3-N4H3F1 (4.07% ± 3.37%) were significantly increased relative to the cNSIP and OP group (p = 0.041 and p = 0.023, respectively). The OP group displayed an intermediate glycosylation profile across the four glycoforms mentioned above. However, OP uniquely exhibited a significantly elevated level of IgG2-N5H4F1 (5.87% ± 1.72%) compared to fNSIP (4.77% ± 1.88%, p = 0.033), indicating a distinct glycosylation feature associated with the OP pattern. These results support IgG glycosylation as a potential marker for classifying IIM-ILD subtypes.

### 3.3 LASSO-weighted multinomial logistic regression for HRCT-defined IIM-ILD subtype prediction

To assess whether the identified glycosylation signatures could effectively discriminate between HRCT-defined IIM-ILD subtypes, we constructed a multinomial logistic regression model incorporating both subclass-specific IGPs and relevant clinical variables. Using one-vs-rest LASSO analysis for feature selection, seven IGPs with non-zero coefficients were retained for modeling: IgG1-N4H4F1, IgG2-N3H4F1, IgG2-N4H4, IgG2-N4H5F1A1, IgG2-N5H3F1, IgG2-N5H4F1, and IgG3-N4H4F1 (**Supplementary Figure 1**). Additionally, seven clinical variables mentioned above that showed statistically significant differences across subtypes were included. Before evaluating the model, we calculated the VIF for each variable and found that all predictors yielded VIF values well under the common threshold of 5.0, suggesting that multicollinearity was not a significant concern in our model (detailed results in **Supplementary Table S4**).To address class imbalance among cNSIP (n = 18), fNSIP (n = 40), and OP (n = 24), we applied inverse class frequency weighting during model training.

**Figure 4:**
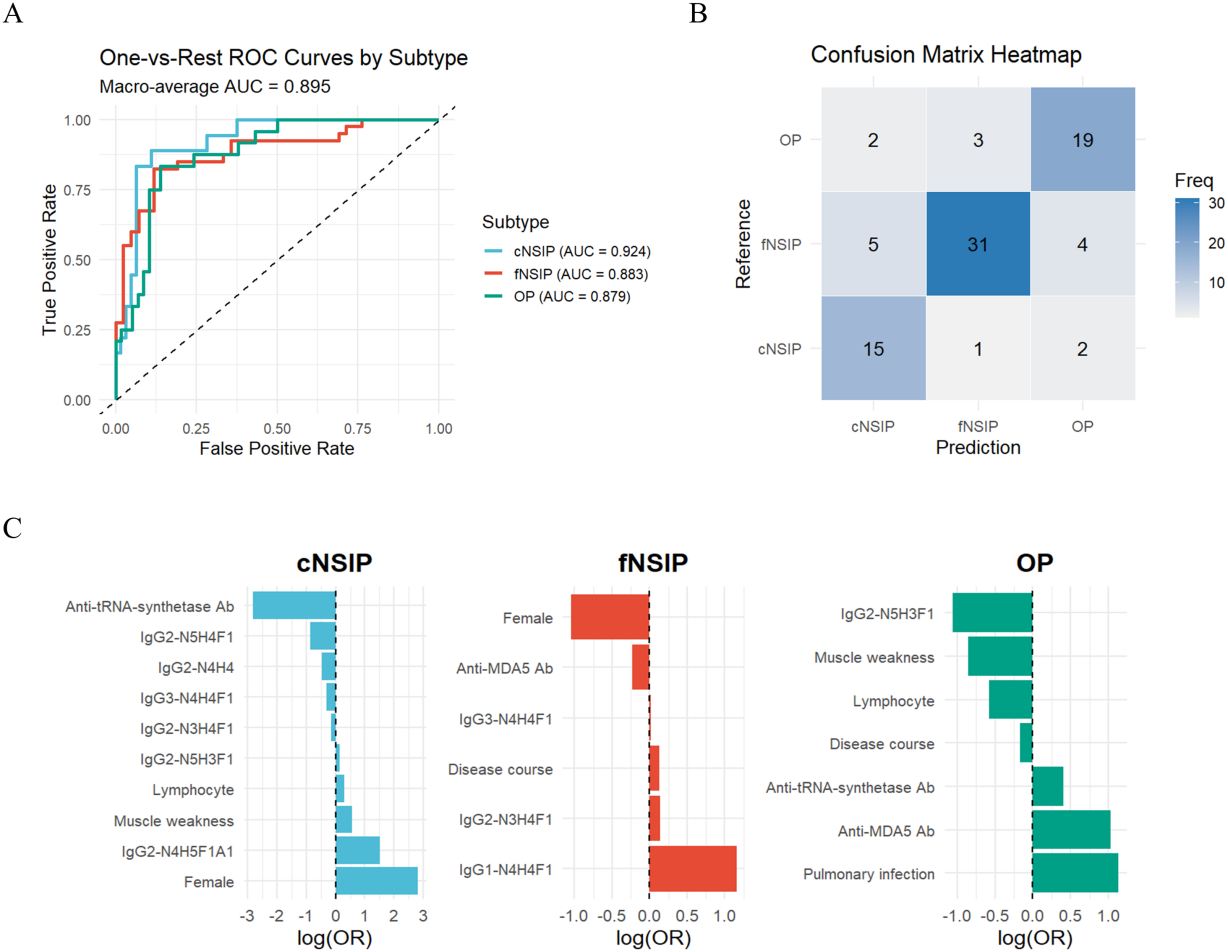
Performance of the weighted multinomial logistic regression model for IIM-ILD subtypes **(A)** Class-specific ROC curves and the macro-averaged AUC of 0.89 for the multinomial logistic regression model distinguishing three HRCT-defined IIM-ILD subtypes. **(B)** Confusion matrix heatmap showing the classification accuracy of the model. **(C)** Bar plot of the regression coefficients (LASSO log-odds ratios) for the predictors retained by the LASSO model, displayed separately for each HRCT subtype. AUC, area under the curve; ROC, receiver operating characteristic; LASSO, least absolute shrinkage and selection operator. cNSIP, cellular nonspecific interstitial pneumonia; fNSIP, fibrotic nonspecific interstitial pneumonia; OP, organizing pneumonia; N, N-acetylhexosamine; H, hexose; F, fucose; A, N-acetylneuraminic acid.

The final model demonstrated strong discriminatory performance across HRCT-defined subtypes, achieving a macro-averaged AUC of 0.89 and a macro-F1 score of 0.78 (**Figure 4A**). Class-specific AUCs were 0.92 for cNSIP, 0.88 for fNSIP, and 0.88 for OP, indicating consistently high predictive accuracy across all categories. The confusion matrix revealed the highest accuracy for fNSIP (31/40 correctly classified), followed by cNSIP (15/18) and OP (19/24) (**Figure 4B**).

The regression coefficients further highlighted subtype-specific predictors (**Figure 4C**). For the cNSIP group, female sex (OR = 16.81), elevated IgG2-N4H5F1A1 (OR = 4.54), and muscle weakness (OR = 1.76) were positively associated, while anti-ARS antibodies positivity was negatively associated (OR = 0.06). In the fNSIP group, key predictors included higher levels of IgG1-N4H4F1 (OR = 3.18) and longer disease duration (OR = 1.14), while anti-MDA5 antibody positivity was inversely correlated (OR = 0.80). In the OP group, anti-MDA5 positivity (OR = 2.79) and pulmonary infection (OR = 3.10) emerged as strong predictors, while IgG2-N5H3F1 (OR = 0.38) and reduced lymphocyte counts (OR = 0.72) were negatively associated. Collectively, these findings suggest that integrating IgG glycosylation profiles with routine clinical parameters enables effective differentiation of HRCT-defined IIM-ILD subtypes.

### 3.4 Correlations between IgG N-glycosylation features and clinical parameters of IIM-ILD patients

**Figure 5:**
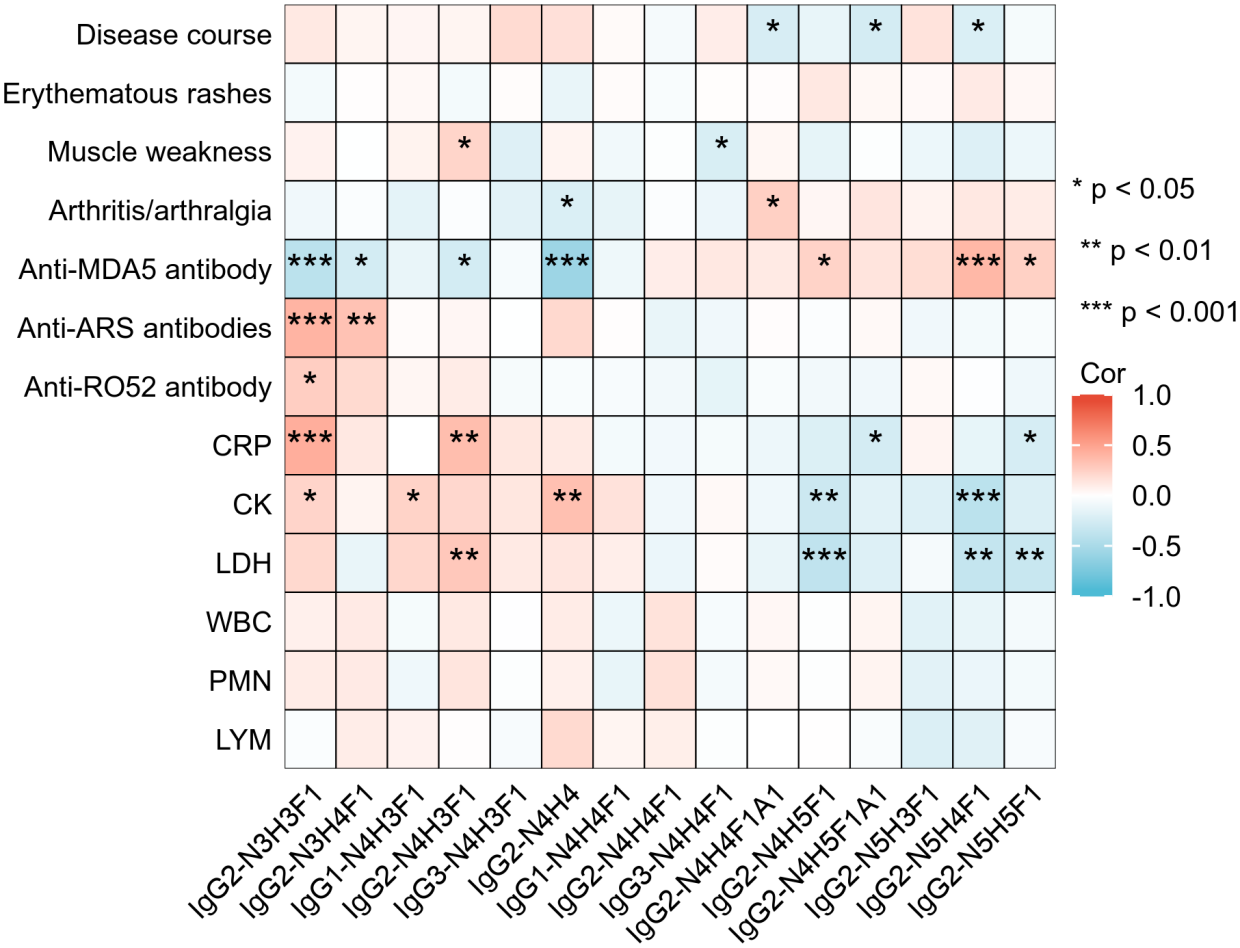
Correlations between IgG N-glycopeptide profiles and clinical features in IIM-ILD patients Heatmap illustrating Spearman correlation coefficients between the relative abundances of 15 subclass-specific intact glycopeptides (IGPs) and selected clinical parameters in patients with IIM-ILD (n = 82). CRP, C-reactive protein; CK, creatine kinase; LDH, lactate dehydrogenase; MB, myoglobin; cTn, cardiac troponin; WBC, white blood cell count; PMN, neutrophil count; LYM, lymphocyte count; ARS, anti-aminoacyl tRNA synthetase antibody; MDA5, melanoma differentiation-associated gene 5 antibody. Asterisks denote statistical significance: *p < 0.05, **p < 0.01, ***p < 0.001.

To enhance the clinical interpretability of IgG subclass-specific glycosylation profiles, we further analyzed their correlations with key clinical indicators in IIM-ILD patients with classified HRCT subtypes (**Figure 5**). Among the IGPs enriched in the IIM-ILD group, IgG2-N4H3F1 correlated positively with muscle weakness (r = 0.23, p = 0.036), CRP (r = 0.36, p = 0.001), and LDH (r = 0.30, p = 0.006), and inversely with anti-MDA5 antibody positivity (r = -0.27, p = 0.015). Conversely, several glycoforms downregulated in IIM-ILD displayed inverse associations. IgG2-N3H4F1 correlated positively with anti-ARS antibody positivity (r = 0.33, p = 0.002) and negatively with anti-MDA5 positivity (r = -0.27, p = 0.015), while IgG2-N4H4 was positively correlated with CK (r = 0.34, p = 0.002) but inversely correlated with anti-MDA5 positivity (r = -0.57, p < 0.001).

Several HRCT subtype-specific glycoforms also showed strong associations. For example, IgG2-N4H5F1A1, enriched in cNSIP patients, was negatively correlated with disease duration (r = –0.26, p = 0.018) and CRP level (r = –0.26, p = 0.019). In the OP subtype, IgG2-N5H4F1 was positively associated with anti-MDA5 positivity (r =0.39, p < 0.001), and negatively correlated with CK (r = –0.41, p < 0.001) and LDH (r = –0.36, p =0.001). In addition to these subtype-defining glycoforms, other IGPs also showed meaningful clinical associations. IgG2-N3H3F1 was positively correlated with CRP (r =0.44, p < 0.001), CK (r =0.23, p =0.036), anti-Ro52 positivity (r =0.27, p = 0.01), and anti-ARS antibodies positivity (r =0.41, p < 0.001), but negatively associated with anti-MDA5 antibody positivity (r = –0.4, p < 0.001). In contrast, IgG2-N4H5F1 and IgG2-N5H5F1 both exhibited a positive association with anti-MDA5 antibody positivity and negative correlations with CRP and LDH.

In addition to these serological parameters, we also identified significant links between IgG glycosylation and pulmonary function (**Supplementary Figure 2**). With regard to ventilatory capacity, IgG2-N4H3F1 was inversely associated with both FVC% (r = –0.27, p = 0.029) and FEV1% (r = –0.25, p = 0.04). Impairment of diffusing capacity was reflected by an inverse correlation between IgG3-N4H3F1 and DLCO% (r = –0.25, p = 0.039), suggesting that this glycoform is associated with more severe interstitial involvement.

## 4 Discussion

Our study systematically profiled subclass-specific IgG N-glycosylation patterns in IIM-ILD patients and evaluated their relationship with HRCT-based imaging subtypes. Using intact glycopeptide profiling, we identified distinct subclass-dependent glycan signatures, especially within IgG2, and demonstrated that their integration with clinical variables enabled robust discrimination of cNSIP, fNSIP, and OP subtypes. These findings suggest that subclass-specific glycosylation may complement radiological assessment and contribute to refined disease endotyping in IIM-ILD patients.

Overall, total sialylation was slightly lower in IIM-ILD. Within IgG2, we observed decreased levels of galactosylated structures, accompanied by an increase in agalactosylated glycoforms, such as IgG2-N4H3F1. These changes align with previous reports showing that lower sialylation enhances the activation of Fcγ receptor engagement, enhancing effector responses such as antibody-dependent cellular cytotoxicity (ADCC) and phagocytosis (20, 21). Similarly, hypogalactosylation of IgG is known to promote immune complex formation and trigger complement activation, contributing to chronic inflammation (22, 23). The global profile in IIM-ILD patients suggests a shift toward a more inflammatory IgG phenotype, which may facilitate pulmonary injury and fibrosis. It is important to recognize the overlap in glycosylation profiles between the IIM-ILD and IIM-NILD patients, as demonstrated in the PCA plot. This is not unexpected, since IIM is a systemic disease and IgG glycosylation reflects a general inflammatory state rather than an organ-specific change. IIM-NILD patients also exhibit subclinical immune activation that precedes detectable lung involvement. Therefore, the overlap probably represents a biological continuum of disease activity. This emphasizes that while broad comparisons are valuable, a more detailed analysis of the patterns within the clinically defined ILD subtypes is essential for identifying signatures with genuine translational potential.

Stratification by HRCT patterns further revealed subtype-specific glycosylation features that paralleled clinical heterogeneity. The cNSIP group was mainly female and had the highest frequency of muscle weakness, while receiving the fewest antifibrotic agents, which is consistent with a more inflammatory and steroid-responsive phenotype reported in the literature (24, 25). Glycomics showed enrichment of galactosylated and sialylated IgG2 glycoforms (e.g., IgG2-N4H5F1A1 and IgG2-N4H4F1), which were negatively correlated with CRP and disease duration. Since both galactosylation and sialylation are known to reduce excessive immune activation and limit effector cell recruitment (20, 23, 26), this glycomics pattern may be associated with a more reversible, immune-driven disease phase rather than an irreversible fibrotic progression. In contrast, fNSIP exhibited longer disease duration and was enriched for anti-ARS positivity, in line with the established link between ASS and NSIP patterns (27). This group exhibited increased glycoformdjs of IgG1 and IgG3, subclasses known for their strong affinity toward C1q and ability to activate Fcγ receptors (6, 28, 29). These changes may reflect enhanced complement-dependent effector activity, and the observed association between agalactosylated IgG3 and reduced diffusing capacity further suggests a potential link between altered glycosylation and impaired pulmonary function. The OP subtype is associated with the shortest disease duration and the highest proportion of anti-MDA5 positivity and pulmonary infection (30). Glycomics revealed increased levels of IgG2-N5H4F1, a glycoform that correlated positively with anti-MDA5 and negatively with CK and LDH levels. IgG2 is usually triggered by high-density polysaccharide antigens and indicates pathogen-driven extrafollicular immune responses (5, 31). However, potential confounding by opportunistic infection at diagnosis cannot be excluded. Our OP glycan signature likely overlaps with influences of infection and underlying autoimmunity rather than a single causal pathway. It is notable that the difference between fNSIP and OP was relatively modest. We propose that this subtle variation in glycan signatures might indicate a shared or transitional immunological state rather than completely separate pathways. This complexity emphasizes the importance of an integrated approach, moving beyond individual markers to a multi-feature model for effective patient stratification.

Building on these subtype-linked glycan signatures, we hypothesized that the potential for clinical translation lies not in any single glycoform, but in an integrated glyo-clinical signature. By combining seven IGPs with seven key clinical factors, our multinomial logistic regression model achieved robust discrimination of HRCT subtypes (macro-AUC = 0.89), indicating translational feasibility. While our cross-sectional design limits definitive conclusions about individual treatment, our findings support translating these signatures into future patient care. First, this approach suggests a potential for refining patient stratification beyond HRCT alone. For example, our results show that the model identified elevated lgG1-N4H4F1, combined with longer disease duration, as strong indicators for fNSIP, which may indicate a more established fibrotic endotype that requires targeted treatment. Second, the model’s subtype-specific predictors enable the development of testable hypotheses for future therapeutic research. The strong link between highly sialylated lgG2-N4H5F1A1 and the cNSIP subtype, which is clinically recognized as more inflammatory, might suggest a higher response to aggressive immunosuppression. Future longitudinal studies are warranted to validate whether monitoring these glyco-clinical signatures can predict disease progression or response to therapy. Consequently, our research offers a foundational framework for the development of tools that may one day assist in customizing management strategies for IIM-ILD.

Several limitations should be acknowledged in this study. First, the single-center design and the relatively small sample size limit the generalizability of our findings. Without an external validation cohort, the accuracy of our predictive model could be overly optimistic, and its effectiveness across wider populations still needs validation. Second, potential confounding by treatment exposure is important. While we found no significant differences in primary therapeutic agents across HRCT subtypes, this does not rule out individual effects of these therapies on IgG glycosylation. Future longitudinal studies should investigate the specific impact of treatments on the glycoprofile. Furthermore, glycan profiles were measured in peripheral blood and may not fully reflect tissue-resident antibody modifications. Thus, the biological links between specific glycoforms and immune effector functions remain associative. Future large-scale, multicenter studies are essential to validate these findings and clarify the mechanistic roles of IgG glycosylation in IIM-ILD.

## 5 Conclusion

This study reveals that subclass-specific IgG N-glycosylation profiles differ across HRCT-defined IIM-ILD subtypes. The integration of glycosylation features with clinical variables can improve the discrimination of fNSIP, cNSIP, and OP phenotypes. These findings highlight the potential value of glycosylation profiling in refining the immunological characterization of IIM-ILD.

## Data Availability

The datasets presented in this study can be found in online repositories. The names of the repository/repositories and accession number(s) can be found in the article/**Supplementary Material**.
