# Supplementary material for: IgG subclass-specific N-glycosylation differentiates HRCT subtypes in idiopathic inflammatory myopathies-associated ILD
Source: Front Immunol. 2025 Nov 3;16:1696126. doi: 10.3389/fimmu.2025.1696126 (PMC12620462; doi:10.3389/fimmu.2025.1696126)
Supplement: Supplementary file 1 [file Table1.docx]

**Supplementary Figure 1.** Subclass-specific IgG N-glycopeptides retained by LASSO for multinomial logistic regression modeling of HRCT subtypes


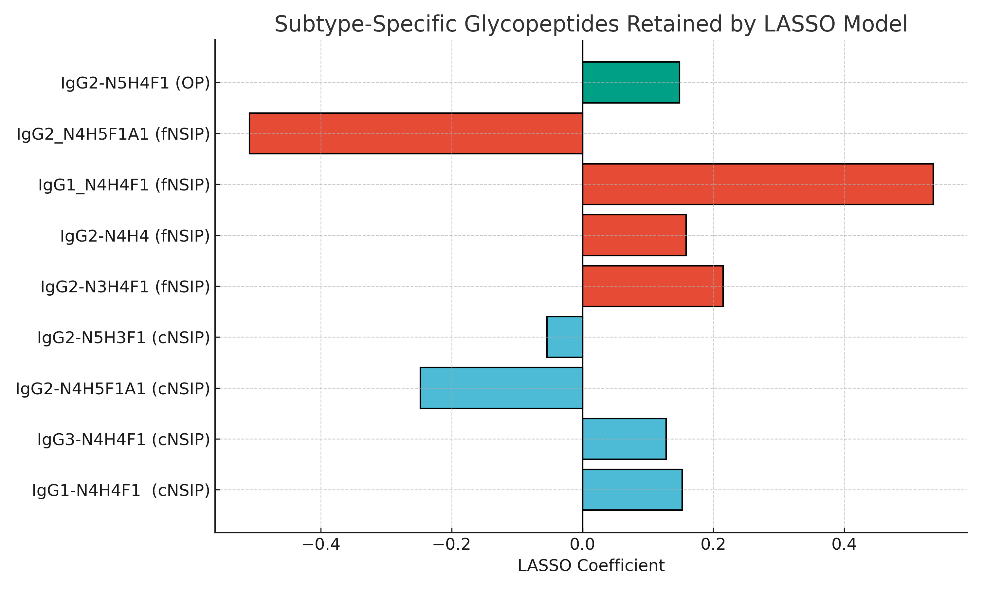


Bar plots displaying the standardized coefficients (λ = λ.min) for seven subclass-specific intact IgG N-glycopeptides (IGPs) retained by the one-vs-rest LASSO analysis. Each bar represents the association between glycopeptide abundance and HRCT-defined IIM-ILD subtypes: cellular NSIP (cNSIP, blue), fibrotic NSIP (fNSIP, red), and organizing pneumonia (OP, green). Positive coefficients indicate a positive correlation with the respective subtype, while negative coefficients indicate an inverse correlation.

IGP, intact glycopeptide; LASSO, least absolute shrinkage and selection operator; cNSIP, cellular nonspecific interstitial pneumonia; fNSIP, fibrotic nonspecific interstitial pneumonia; OP, organizing pneumonia.

**Supplementary Figure 2.** Correlation between IgG N-glycopeptide profiles and pulmonary function parameters in IIM-ILD patients


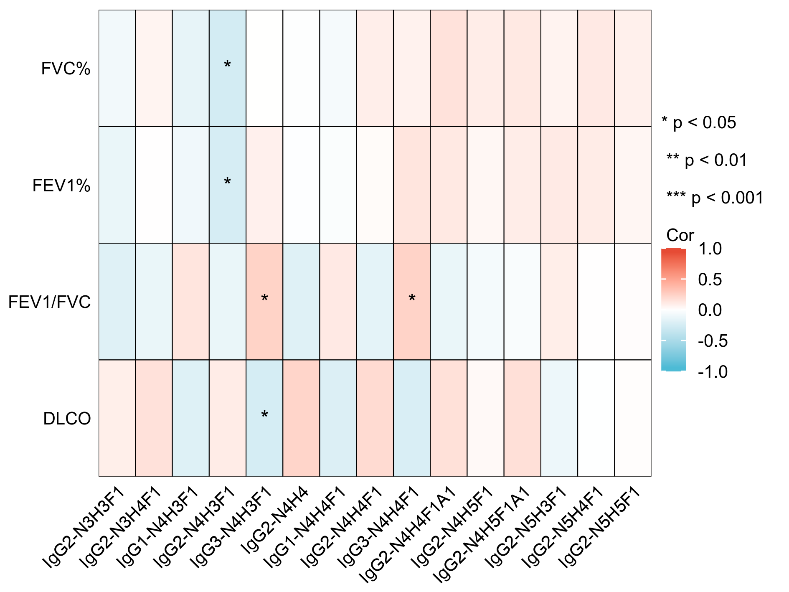


Heatmap displaying correlations between IgG subclass-specific N-glycopeptides and pulmonary function indices (FVC%, FEV1%, FEV1/FVC ratio, and DLCO%, n=57). Correlation coefficients were calculated using Spearman’s rank test. Color intensity reflects the strength and direction of correlation, with red indicating positive and blue indicating negative associations. N, N-acetylhexosamine; H, hexose; F, fucose; A, N-acetylneuraminic acid. Significant correlations are marked with * p < 0.05.
