# Supplementary material for: IgG subclass-specific N-glycosylation differentiates HRCT subtypes in idiopathic inflammatory myopathies-associated ILD
Source: Front Immunol. 2025 Nov 3;16:1696126. doi: 10.3389/fimmu.2025.1696126 (PMC12620462; doi:10.3389/fimmu.2025.1696126)
Supplement: Supplementary file 2 [file Table2.docx]

Supplementary Material

**Supplementary Table 1.** Baseline Characteristics of the IIM Patients Stratified by ILD Status

| **Variables** | **IIM-ILD**  **(n=98)** | **IIM-NILD**  **(n=47)** | **P value** |
| --- | --- | --- | --- |
| Demographic data |  |  |  |
| Female, n (%) | 74 (75.51) | 40 (85.11) | 0.2036 |
| Age, years (mean, SD) | 49.07 (11.26) | 47.40 (12.51) | 0.3163 |
| BMI, kg/m^2^ (mean, SD) | 22.25 (3.11) | 22.81 (3.54) | 0.3343 |
| Disease duration,  months (median, IQR) | 6.0 (2.5, 19.5) | 9.0 (2.0, 54.0) | 0.7027 |
| Smoking history, n (%) | 11 (11.22) | 4 (8.51) | 0.7743 |
| Diagnosis, n (%) |  |  |  |
| ADM | 4 (16.67) | 20 (83.33) | <0.0001 |
| ASS | 33 (94.29) | 2 (5.71) | <0.0001 |
| DM | 37 (59.68) | 25 (40.32) | 0.1062 |
| IMNM | 24 (100.0) | - | <0.0001 |
| Clinical manifestations, n (%) |  |  |  |
| Erythematous rashes | 40 (40.82) | 21 (44.68) | 0.7206 |
| Muscle weakness | 31 (31.63) | 32 (68.09) | <0.0001 |
| Arthritis/arthralgia | 28 (28.57) | 7 (14.89) | 0.0967 |
| Laboratory findings |  |  |  |
| RO52 (+), n (%) | 62 (63.27) | 13 (27.66) | <0.0001 |
| ARS (+), n (%) | 33 (33.67) | 3 (6.38) | 0.0002 |
| MDA5 (+), n (%) | 52 (53.06) | 12 (25.53) | 0.0023 |
| IMNM-associated MSAs (+)^†^, n (%) | 5 (5.10) | 9 (19.15) | 0.0135 |
| CRP, ng/ml (median, IQR) | 4.58 (2.15, 9.33) | 2.89 (1.79, 5.43) | 0.0715 |
| CK, IU/L (median, IQR) | 58.00 (28.00, 315.0) | 240.0 (86.00, 1924) | <0.0001 |
| LDH, IU/L (median, IQR) | 271.0 (230.3, 357.3) | 375.0 (235.0, 587.0) | 0.0200 |
| WBC, ×10^9^/L (mean, SD) | 8.12 (3.83) | 6.88 (3.33) | 0.0600 |
| PMN, ×10^9^/L (mean, SD) | 6.08 (3.36) | 4.69 (2.44) | 0.0056 |
| LYM, ×10^9^/L (mean, SD) | 1.35 (0.75) | 1.52 (0.88) | 0.2242 |

†: IMNM-associated MSAs included anti-SRP and anti-HMGCR antibodies. IIM, idiopathic inflammatory myopathies; ILD, interstitial lung disease; BMI, body mass index; ADM, amyopathic dermatomyositis; DM, dermatomyositis; ASS, anti-synthetase syndrome; IMNM, immune-mediated necrotizing myopathy; ARS, anti-aminoacyl tRNA synthetase antibodies; MDA5, melanoma differentiation-associated protein 5 antibody; MSAs, myositis-specific antibodies; MAAs, myositis-associated antibodies; CRP, C-reactive protein; CK, creatine kinase; LDH, lactate dehydrogenase; WBC, white blood cells; PMN, polymorphonuclear neutrophils; LYM, lymphocytes

**Supplementary Table 2.** The medication regimen across HRCT-defined subtypes

| **Medication** | **cNSIP**  **(n=18)** | **fNSIP**  **(n=40)** | **OP**  **(n=24)** | **P value** |
| --- | --- | --- | --- | --- |
| Treatment naïve, n (%) | 7 (38.89) | 12 (30.00) | 6 (25.00) | 0.6057 |
| Treatment during this admission, n (%) |  |  |  |  |
| GC <50mg/d | 6 (33.33) | 24 (60.00) | 11 (45.83) | 0.1693 |
| GC ≥50mg/d | 1 (5.56) | 2 (5.00) | 5 (20.83) | 0.1359 |
| HCQ | 1 (5.56) | 5 (12.50) | 3 (12.50) | 0.8086 |
| CTX | 3 (16.67) | 6 (15.00) | 4 (16.67) | >0.9999 |
| CNI | 5 (27.78) | 9 (22.50) | 2 (8.33) | 0.1954 |
| Other csDMARDs^¶^ | 1 (5.56) | 3 (7.50) | 1 (4.17) | >0.9999 |
| JAK inhibitors^¶¶^ | 2 (11.11) | 3 (7.50) | - | 0.2362 |
| Anti-pulmonary fibrosis drugs^¶¶¶^ | - | 9 (22.50) | 3 (12.50) | 0.0749 |

¶: Other csDMARDs included methotrexate and mycophenolate mofetil. ¶¶: JAK inhibitors included baricitinib and tofacitinib. ¶¶¶: Anti-pulmonary fibrosis drugs included nintedanib and pirfenidone. GC, glucocorticoids; HCQ, hydroxychloroquine; CTX, cyclophosphamide; CNI, calcineurin inhibitor; csDMARDs, conventional synthetic disease-modifying anti-rheumatic drugs.

| **IGPs**  **(mean, SD)** | **IIM-ILD**  **(n=24)** | **IIM-NILD**  **(n=98)** | **P value** |
| --- | --- | --- | --- |
| Core fucosylation | 99.43 (0.42) | 99.16 (0.47) | 0.0003 |
| Sialylation | 7.23 (3.00) | 8.19 (2.73) | 0.0461 |
| IgG1-N4H3F1 | 4.34 (3.51) | 3.26 (2.27) | 0.2300 |
| IgG1-N4H4F1 | 4.39 (3.36) | 4.67 (2.93) | 0.2472 |
| IgG2-N3H3F1 | 3.38 (1.20) | 3.04 (0.78) | 0.0851 |
| IgG2-N3H4F1 | 1.00(0.36) | 1.15(0.34) | 0.0233 |
| IgG2-N4H3F1 | 27.93(6.52) | 24.31(6.11) | 0.0017 |
| IgG2-N4H4 | 0.57(0.42) | 0.84(0.47) | 0.0007 |
| IgG2-N4H4F1 | 22.86(4.60) | 26.27(4.70) | <0.0001 |
| IgG2-N4H4F1A1 | 3.08(1.03) | 3.48(0.93) | 0.0253 |
| IgG2-N4H5F1 | 7.85(3.91) | 8.44(3.25) | 0.3762 |
| IgG2-N4H5F1A1 | 4.15(2.21) | 4.71(2.11) | 0.1522 |
| IgG2-N5H3F1 | 7.11(1.99) | 5.83(1.93) | 0.0004 |
| IgG2-N5H4F1 | 5.19(1.85) | 5.37(2.17) | 0.6052 |
| IgG2-N5H5F1 | 0.94(0.61) | 1.08(0.60) | 0.2084 |
| IgG3-N4H3F1 | 3.49(2.92) | 3.35(2.56) | 0.6932 |
| IgG3-N4H4F1 | 3.71(2.91) | 4.20(3.07) | 0.3529 |

**Supplementary Table 3.** Differential abundance of intact IgG N-Glycopeptides between IIM-ILD and IIM-NILD patients

Note: IGPs, Intact N-glycopeptides; N: N-acetylhexosamine; H: hexose; F: fucose; A: N-acetylneuraminic acid.

**Supplementary Table 4.** Results of multicollinearity diagnostics for predictors in the multinomial logistic regression model

| **Predictor Variable** | **Variance Inflation Factor (VIF)** |
| --- | --- |
| IgG1-N4H4F1 | 1.96 |
| IgG3-N4H4F1 | 2.13 |
| IgG2-N4H5F1A1 | 2.54 |
| IgG2-N5H3F1 | 2.06 |
| IgG2-N5H4F1 | 2.72 |
| IgG2-N3H4F1 | 1.93 |
| IgG2-N4H4 | 1.90 |
| Female | 1.16 |
| Muscle weakness | 1.25 |
| Anti-MDA5 antibody | 3.85 |
| Anti-tRNA synthetase antibodies | 2.40 |
| Pulmonary infection | 1.25 |
| Lymphocyte | 1.28 |

The variance inflation factor (VIF) was calculated for each of the 14 predictor variables included in the final model. A VIF value < 5.0 is generally considered to indicate a low level of multicollinearity.
